# Supplementary material for: Neutralization of SARS-CoV-2 Omicron BQ.1, BQ.1.1 and XBB.1 variants following SARS-CoV-2 infection or vaccination in children
Source: Nat Commun. 2023 Dec 1;14:7952. doi: 10.1038/s41467-023-43152-y (PMC10692185; doi:10.1038/s41467-023-43152-y)
Supplement: Supplementary file 1 — Supplementary Information [file 41467_2023_43152_MOESM1_ESM.pdf]

SUPPLEMENTAL INFORMATION (Neutralization of SARS-CoV-2 Omicron BQ.1, BQ.1.1 and XBB.1 variants following SARS-CoV-2 infection or vaccination in children)

Overcoming COVID-19 Investigators Consortium

The following non-authors contributed to consenting and enrolling patients as well as collecting samples and clinical data for this study.

Suden Kucukak, MD[5], Margaret M. Newhams, MPH[5], Elizabeth R. McNamara BSN, RN[5], Marah Kiana Echon Rosales, BS[5], Jeni Melo, BS[5], Hye Kyung Moon, MA[5], Sergio R. Jackson, MS[5], Sabrina R. Chen, BS[5], Janet Chou, MD[6], Megan Elkins, MHS[6], Michele Kong, MD[7], Ronald C. Sanders Jr., MD, MS[8], Katherine Irby, MD[8], Natalie Z. Cvijanovich, MD[9], Matt S. Zinter, MD[10], Aline B. Maddux, MD, MSCS[11],Christina M. Osborne, MD[11], Sara Shankman, DNP, CPNC-AC[11], Emily Port, BA, PMP[11], Rachel Mansour, BSN, RN, CPN[11], Natasha Baig, MBBS[11], Frances Zorensky, BS[11], Keiko M. Tarquinio, MD[12], Kaitlin Jones, MSN, RN, CCRP[12], Kelly N. Michelson, MD, MPH[13], Bria M. Coates, MD[13], Courtney M. Rowan, MD, MScr[14], Heidi R. Flori, MD, FAAP[15], Mary K. Dahmer, PhD[15], Janet R. Hume, MD, PhD[16], Charlotte V. Hobbs, MD[17], Lora Martin, NP[17], Lacy Malloch, BS[17], John M. Williams, MS[17], Gurbaksh Singh, MS[17], Urita Agana, BS[17], Kayla Patterson, MS[17], Jennifer E. Schuster, MD[18], Abigail Kietzman BS[18], Shannon Hill, BSN[18], Melissa L. Cullimore MD, PhD[19], Russell J. McCulloh, MD[19], Shira J. Gertz, MD[20], Stephanie P. Schwartz, MD[21], Tracie C. Walker, MD[21], Steven L. Shein, MD[22], Amanda N. Lansell, MD[22] Mary A. Staat, MD, MPH[23], Chelsea C. Rohlfs, BS, MBA[23], Julie C. Fitzgerald, MD, PhD, MSCE[24], Jenny L. Bush RN, BSN[24], Ryan H. Burnett, BS[24], Elizabeth H. Mack, MD, MS[25], Nelson Reed, MD[25] , Laura Smallcomb, MD[25], Natasha B. Halasa, MD, MPH26, Laura L. Loftis, MD[27], Hillary Crandall, MD PhD[28], Kwabena Krow Ampofo, MD[28]

[5] Department of Anesthesiology, Critical Care and Pain Medicine, Boston Children's Hospital, Boston, MA, USA.

[6] Division of Immunology, Boston Children's Hospital and Harvard Medical School, Boston, MA, USA.

[7] Children's of Alabama Division of Pediatric Critical Care Medicine, Department of Pediatrics, University of Alabama at Birmingham, Birmingham, AL, USA.

[8] Section of Critical Care, Department of Pediatrics, Arkansas Children's Hospital, Little Rock, AR, USA.

[9] Division of Critical Care Medicine, UCSF Benioff Children's Hospital Oakland, CA, USA.

[10] Department of Pediatrics, Divisions of Critical Care and Bone Marrow Transplantation, University of California, San Francisco Benioff Children's Hospital, San Francisco, CA, USA.

[11] Department of Pediatrics, Section of Critical Care Medicine, University of Colorado School of Medicine and Children's Hospital Colorado, Aurora, CO, USA.

[12] Division of Critical Care Medicine, Department of Pediatrics, Emory University School of Medicine, Children's Healthcare of Atlanta at Egleston, Atlanta, GA, USA.

[13] Division of Critical Care Medicine, Department of Pediatrics, Northwestern University Feinberg School of Medicine, Ann & Robert H. Lurie Children's Hospital of Chicago, Chicago, IL, USA.

[14] Division of Pediatric Critical Care Medicine, Department of Pediatrics, Indiana University School of Medicine, Riley Hospital for Children, Indianapolis, IN, USA.

[15] Division of Pediatric Critical Care Medicine, Department of Pediatrics, University of Michigan CS Mott Children's Hospital, Ann Arbor, MI, USA.

[16] Division of Pediatric Critical Care, University of Minnesota Masonic Children's Hospital, Minneapolis, MN, USA.

[17] Department of Pediatrics, Division of Infectious Diseases, University of Mississippi Medical Center, Jackson, MS, USA.

[18] Division of Pediatric Infectious Diseases, Department of Pediatrics, Children's Mercy Kansas City, Kansas City, MO, USA.

[19] Division of Pediatric Critical Care, Department of Pediatrics, Children's Hospital and Medical Center, Omaha, NE, USA.

[20] Division of Pediatric Critical Care, Department of Pediatrics, Cooperman Barnabas Medical Center, Livingston, NJ, USA.

[21] Department of Pediatrics, University of North Carolina at Chapel Hill Children's Hospital, Chapel Hill, NC, USA.

[22] Division of Pediatric Critical Care Medicine, University Hospitals Rainbow Babies and Children's Hospital, Cleveland, OH, USA.

[23] Department of Pediatrics, University of Cincinnati, Division of Infectious Diseases, Cincinnati Children's Hospital Medical Center, Cincinnati, OH, USA.

[24] Division of Critical Care, Department of Anesthesiology and Critical Care, The University of Pennsylvania Perelman School of Medicine, Children's Hospital of Philadelphia, Philadelphia, PA, USA.

[25] Division of Pediatric Critical Care Medicine, Medical University of South Carolina Children's Health, Charleston, SC, USA.

[26] Division of Pediatric Infectious Diseases, Department of Pediatrics, Monroe Carell Jr. Children's Hospital at Vanderbilt, Vanderbilt University Medical Center, Nashville, TN, USA.

[27] Section of Critical Care Medicine, Department of Pediatrics, Baylor College of Medicine, Texas Children's Hospital, Houston, TX, USA.

[28] Division of Pediatric Critical Care, Department of Pediatrics, University of Utah Primary Children's Hospital, Salt Lake City, UT, USA.

Taking on COVID-19 Together Team

The following non-authors contributed to consenting and enrolling patients as well as collecting samples and clinical data for this study.

Suden Kucukak, MD[4], Margaret M. Newhams, MPH[4], Elizabeth R. McNamara BSN, RN[4], Marah Kiana Echon Rosales, BS[4], Jeni Melo, BS[4], Hye Kyung Moon, MA[4], Sergio R. Jackson, MS[4], Sabrina R. Chen, BS[4], Janet Chou, MD[6], Megan Elkins, MHS[6], David Williams, MD[29], Lucinda Williams, DNP, MSN, RN, PNP, NE-BC[29], Leah Cheng, MA[29], Yubo Zhang, BS[29], Danielle Crethers, BA[29], Debra Morley, PhD[29], Sarah Steltz, MPH[29], Kelly Zakar, MSN, RN, PPCNP-BC[29], Kristin Moffitt, MD[30], Myriam A. Armant, PhD[31], Felicia Ciuculescu, MD[31]

[4] Department of Anesthesiology, Critical Care and Pain Medicine, Boston Children's Hospital, Boston, MA, USA.

[6] Division of Immunology, Boston Children's Hospital, Harvard Medical School, Boston, MA, USA.

[29] Institutional Centers for Clinical and Translational Research, Boston Children's Hospital, Boston, MA, USA.

[30] Pediatric Infectious Diseases Division, Boston Children's Hospital, Harvard Medical School, Boston, MA, USA.

[31] TransLab Core, Boston Children's Hospital, Boston, MA, USA.

Table S1: SARS-CoV-2 variants mutations introduced in the spike plasmid for production of SARS-CoV-2 pseudovirions for analysis in PsVNA.

SARS-CoV-2 variant	Mutations constructed in the spike plasmids
Omicron (BA.2)	T19I, delL24, delP25, delP26, A27S, G142D, V213G, G339D, S371F, S373P, S375F, T376A, D405N, R408S, K417N, N440K, S477N, T478K, E484A, Q493R, Q498R, N501Y, Y505H, D614G, H655Y, N679K, P681H, N764K, D796Y, Q954H, N969K
Omicron (BA.2.75)	BA.2 spike mutations (T19I, delL24, delP25, delP26, A27S, G142D, V213G, G339D, S371F, S373P, S375F, T376A, D405N, R408S, K417N, N440K, S477N, T478K, E484A, Q493R, Q498R, N501Y, Y505H, D614G, H655Y, N679K, P681H, N764K, D796Y, Q954H, N969K) + K147E, W152R, F157L, I210V, G257S, D339H, G446S, N460K, Q493reversion
Omicron (BA.3)	A67V, del69-70, del142-144, Y145D, del211, L212I, G339D, S371F, S373P, S375F, D405N, K417N, N440K, G446S, S477N, T478K, E484A, Q493R, Q498R, N501Y, Y505H, D614G, H655Y, N679K, P681H, N764K, D796Y, Q954H, N969K
Omicron (BA.4/BA.5)	T19I, delL24, delP25, delP26, A27S, del69 70, G142D, V213G, G339D, S371F, S373P, S375F, T376A, D405N, R408S, K417N, N440K, L452R, S477N, T478K, E484A, F486V, Q498R, N501Y, Y505H, D614G, H655Y, N679K, P681H, N764K, D796Y, Q954H, N969K
Omicron (BQ.1)	BA.4/BA.5 spike mutations (T19I, delL24, delP25, delP26, A27S, del69 70, G142D, V213G, G339D, S371F, S373P, S375F, T376A, D405N, R408S, K417N, N440K, L452R, S477N, T478K, E484A, F486V, Q498R, N501Y, Y505H, D614G, H655Y, N679K, P681H, N764K, D796Y, Q954H, N969K) + K444T and N460K.
Omicron (BQ.1.1)	BQ.1 spike mutations (T19I, delL24, delP25, delP26, A27S, del69 70, G142D, V213G, G339D, S371F, S373P, S375F, T376A, D405N, R408S, K417N, N440K, L452R, S477N, T478K, E484A, F486V, Q498R, N501Y, Y505H, D614G, H655Y, N679K, P681H, N764K, D796Y, Q954H, N969K, K444T and N460K) + R346T.
Omicron (XBB.1)	BA.2 spike mutations (T19I, delL24, delP25, delP26, A27S, G142D, V213G, G252V, G339D, S371F, S373P, S375F, T376A, D405N, R408S, K417N, N440K, S477N, T478K, E484A, Q493R, Q498R, N501Y, Y505H, D614G, H655Y, N679K, P681H, N764K, D796Y, Q954H, N969K) + V83A, Del144, H146Q, Q183E, V213E, G339H, R346T, L368I, V445P, G446S, N460K, F486S, F490S, R493Q

Table S2: Patient's demographic data

Demographics Table	UNVACCINATED									VACCINATED		
Category	Acute COVID-19 (n=54)			MIS-C (n=64)			Conval. Outpatients (n=33)			Healthy controls (n=62)		
Age Group	<5	5 to <12	12 to 21	<5	5 to 11	12 to 21	<5	5 to 11	12 to 21	<5	5 to 11	12 to 21
Age (yrs): median (IQR)	0.5 (0.13, 1.2)	8.7 (7.8, 9)	16 (15, 17.8)	3 (1.5, 4.2)	8.2 (7.3, 10)	14.9 (14, 17.1)	1.6 (1, 2.5)	8.4 (6.2, 9.5)	16.6 (13.3, 19)	2 (1, 3)	9 (8, 10)	14 (12.5, 15)
N	23	10	21	22	18	24	10	11	12	10	32	20
Sex: Male n (%)	12 (52.2)	6 (60)	8 (38.1)	10 (45.5)	11 (61.1)	16 (66.7)	3 (30)	8 (72.7)	9 (75)	6 (60%)	16 (50)	7 (35)
Hispanic or Latino n (%)	9 (39.1)	4 (40)	9 (42.9)	9 (40.9)	7 (38.9)	5 (20.8)	4 (40)	4 (36.4)	4 (33.3)	2 (20%)	2 (6.3)	5 (25)
White or White-Hispanic n (%)	13 (56.5)	5 (50)	10 (47.6)	10 (45.5)	9 (50)	15 (62.5)	3 (30)	1 (9.1)	4 (33.3)	5 (50%)	25 (78.1)	12 (60)
Black, African American or Black-Hispanic n (%)	5 (21.7)	2 (20)	4 (19)	5 (22.7)	3 (16.7)	7 (29.2)	0	0	1 (8.3)	0	see Other	see Other
Asian n (%)	0	0	1 (4.8)	1 (4.5)	0	1 (4.2)	0	0	1 (8.3)	1 (10%)	3 (9.4)	1 (5)
Other or Unknown n (%)	5 (21.7)	3 (30)	6 (28.6)	6 (27.3)	6 (33.3)	1 (4.2)	7 (70)	10 (90.9)	6 (50)	2 (20%)	4 (12.5)	7 (35)
Previously healthy n (%)	16 (69.6)	3 (30%)	3 (14.3)	20 (90.9)	10 (55.6)	13 (54.2)	6 (60)	4 (36.4)	5 (41.7)	10	32 (100)	20 (100)
Day of research blood collection* Median (IQR)	1 (1, 2)	1 (1, 2.5)	2 (1, 4)	3 (1.3, 6)	2.5 (1, 5)	4 (2, 14.3)	60.5 (42.5, 80)	76 (60.5, 85)	69 (61.3, 74.3)	20 (18, 22)	19.5 (15, 30.8)	16.5 (15, 18)

* Number of days after: PCR or Ab positive test (for Acute COVID-19 inpatients), PCR positive test (for convalescent outpatients), hospitalization (for MIS-C), or vaccination (for controls) that research blood sample was collected. When comparing the three age groups within each disease category using the Kruskal-Wallis test, there were no statistical differences in the timing of pediatric blood sample collection relative to the PCR/antibody positive test date for acute and convalescent patients or hospital admission date for MIS-C patients.

Figure S1

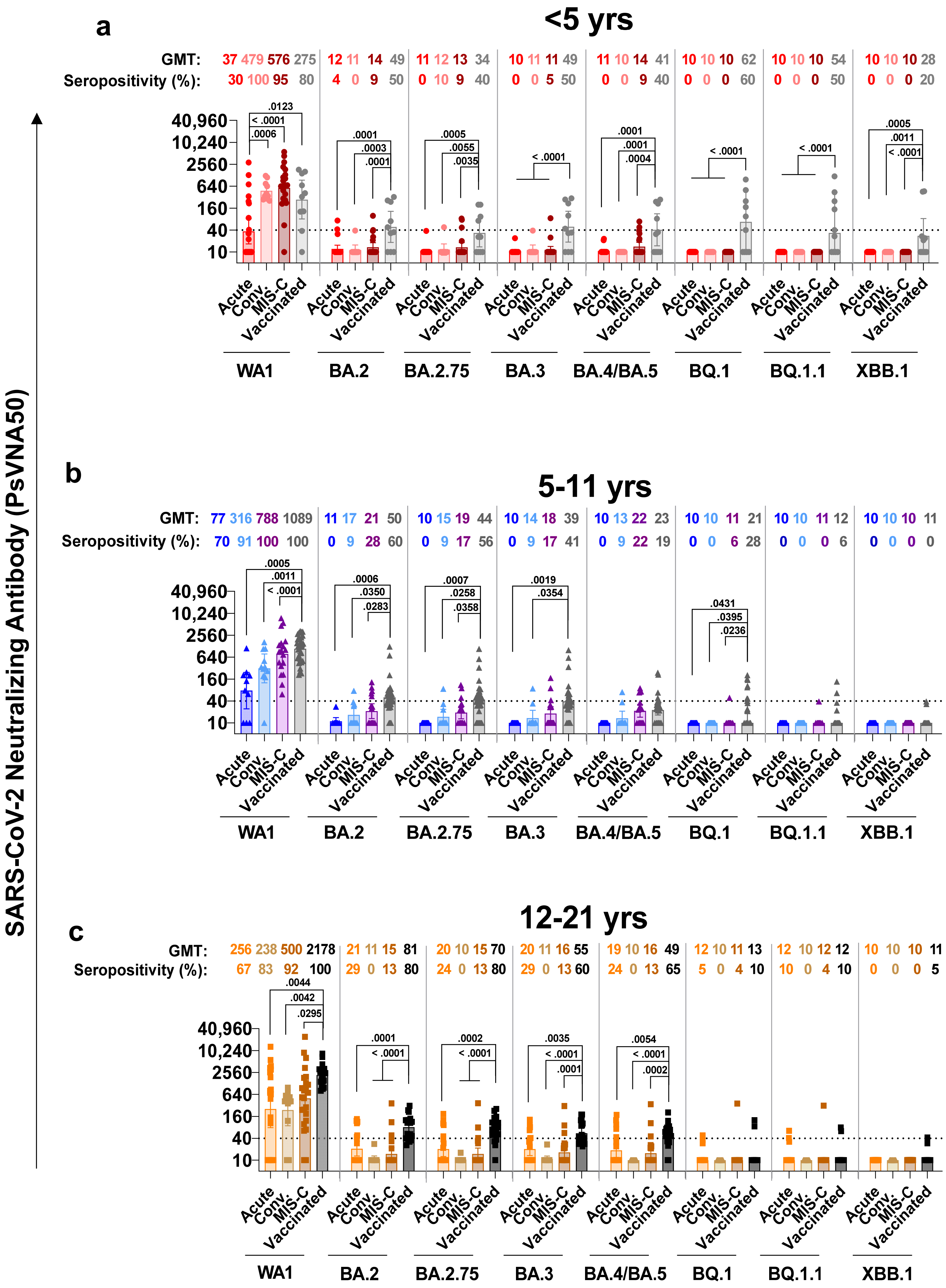

SARS-CoV-2 Neutralizing Antibody (PsVNA50)
a
<5 yrs
GMT: 37 479 576 275 | 12 11 14 49 | 11 12 13 34 | 10 11 11 49 | 11 10 14 41 | 10 10 10 62 | 10 10 10 54 | 10 10 10 28
Seropositivity (%): 30 100 95 80 | 4 0 9 50 | 0 10 9 40 | 0 0 5 50 | 0 0 9 40 | 0 0 0 60 | 0 0 0 50 | 0 0 0 20
.0123
< .0001
.0006
.0001
.0003
.0001
.0005
.0055
.0035
< .0001
.0001
.0001
.0004
< .0001
< .0001
.0005
.0011
< .0001
40,960
10,240
2560
640
160
40
10
Acute
Conv.
MIS-C
Vaccinated
WA1
BA.2
BA.2.75
BA.3
BA.4/BA.5
BQ.1
BQ.1.1
XBB.1
b
5-11 yrs
GMT: 77 316 788 1089 | 11 17 21 50 | 10 15 19 44 | 10 14 18 39 | 10 13 22 23 | 10 10 11 21 | 10 10 11 12 | 10 10 10 11
Seropositivity (%): 70 91 100 100 | 0 9 28 60 | 0 9 17 56 | 0 9 17 41 | 0 9 22 19 | 0 0 6 28 | 0 0 0 6 | 0 0 0 0
.0005
.0011
< .0001
.0006
.0350
.0283
.0007
.0258
.0358
.0019
.0354
.0431
.0395
.0236
c
12-21 yrs
GMT: 256 238 500 2178 | 21 11 15 81 | 20 10 15 70 | 20 11 16 55 | 19 10 16 49 | 12 10 11 13 | 12 10 12 12 | 10 10 10 11
Seropositivity (%): 67 83 92 100 | 29 0 13 80 | 24 0 13 80 | 29 0 13 60 | 24 0 13 65 | 5 0 4 10 | 10 0 4 10 | 0 0 0 5
.0044
.0042
.0295
.0001
< .0001
.0002
< .0001
.0035
< .0001
.0001
.0054
< .0001
.0002

Figure S1: Comparison of neutralizing antibodies in different age group children with acute COVID-19 vs convalescent COVID-19 vs MIS-C vs vaccination against SARS-CoV-2 WA-1 and Omicron subvariants.

SARS-CoV-2 neutralization assays were performed using pseudoviruses expressing the spike protein of WA-1/2020 or the Omicron subvariants BA.2, BA.2.75, BA.3, BA.4/BA.5, BQ.1, BQ.1.1 and XBB.1, in 293-ACE2-TMPRSS2 cells. Samples were divided by age categories: <5 years (n=65; 23 acute, 10 convalescent, 22 MIS-C and 10 naïve vaccinated), 5-11 years (n=71; 10 acute, 11 convalescent, 18 MIS-C and 32 naïve vaccinated) and 12-21 years old (n=77; 21 acute, 12 convalescent, 24 MIS-C and 20 naïve vaccinated). Geometric mean titer (GMT) values ± 95% CI of PsVNA50 (50% neutralization) titers for samples from youngest children (<5 years; in a), 5-11 years (b), and adolescent (12-21 years, in c), with either acute COVID-19 (in red), convalescent COVID-19 (in orange), MIS-C (in burgundy), or mRNA vaccination (in black) are shown. All PsVNA experiments were performed twice and the researchers performing the assay were blinded to sample identity. The variations for duplicate runs was <6%. The data shown are average values of two experimental runs. Statistical differences were analyzed in R and the two-sided statistically significant p-values are shown.

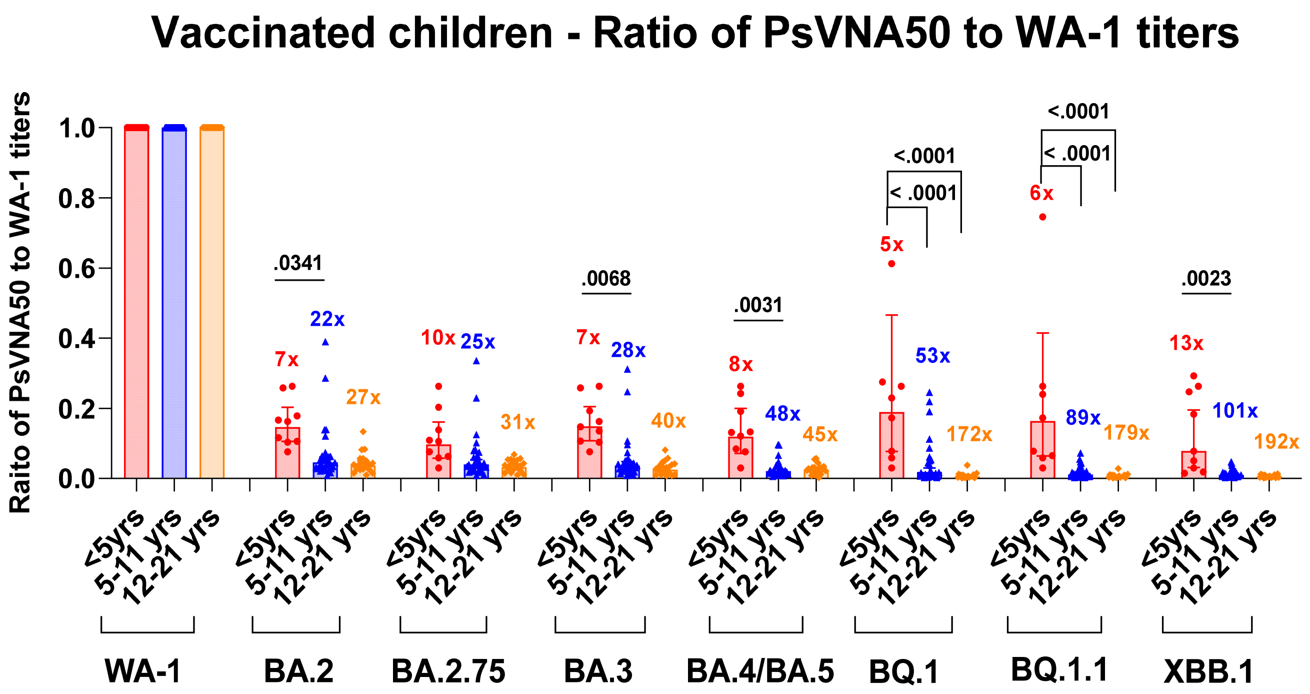

Figure S2: Reduction in capacity of neutralizing antibodies following vaccination against SARS-CoV-2 Omicron subvariants compared with WA-1 in different pediatric age groups.

SARS-CoV-2 neutralization assays were performed using pseudoviruses expressing the spike protein of WA-1/2020 or the Omicron subvariants BA.2, BA.2.75, BA.3, BA.4/BA.5, BQ.1, BQ.1.1 and XBB.1, in 293-ACE2-TMPRSS2 cells. Samples from children vaccinated with mRNA vaccines were divided into age categories: <5 years (n=10; red), 5-11 years (n=32; blue) and 12-21 years old (n=20; orange). A ratio was calculated by dividing the PsVNA50 titers against Omicron subvariants by the corresponding PsVNA50 titers against the vaccine-homologous prototype WA-1 strain for every individual. All PsVNA experiments were performed twice and the researchers performing the assay were blinded to sample identity. The variations for duplicate runs was <6%. The data shown are average values of two experimental runs. Statistical differences were analyzed in R and the two-sided statistically significant p-values are shown.

Figure S3

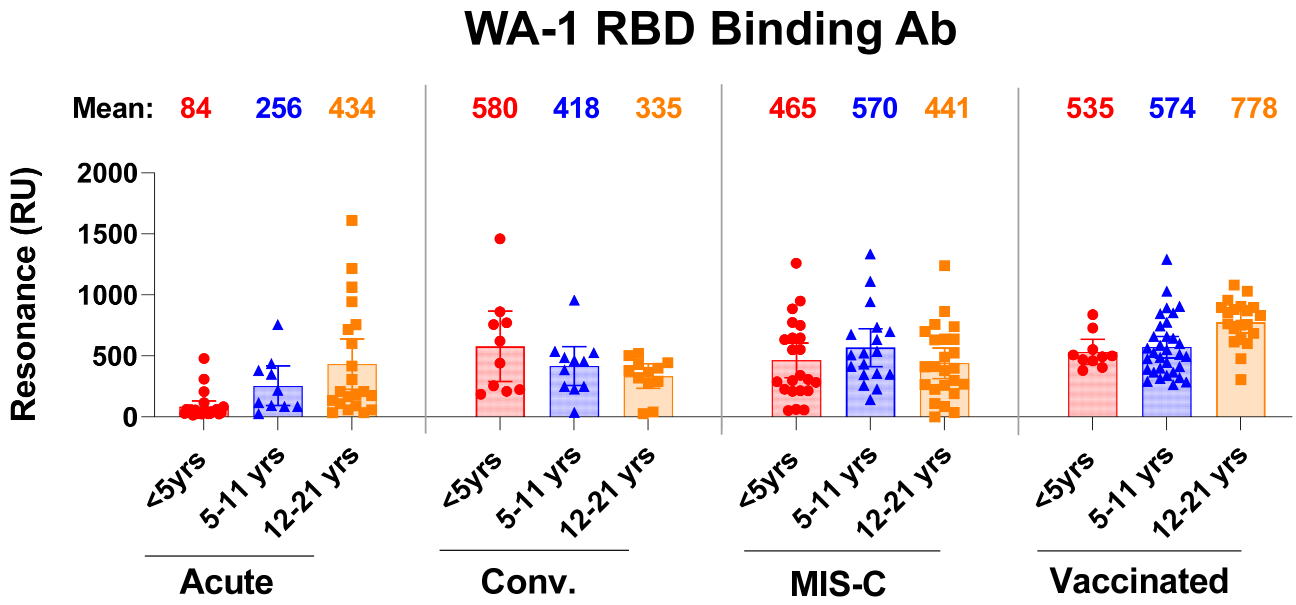

Figure S3. Binding antibodies in serum/plasma of children with COVID-19 vs MIS-C vs vaccination to the receptor binding domain of spike protein from prototype vaccine-homologous SARS-CoV-2 WA-1 strain.
Total antibody binding (determined by maximum resonance units, Max RU) of 1:10 diluted serum or plasma to purified WA-1 RBD was measured by SPR. Data is shown either by infection or vaccination category. Samples were divided by age categories: <5 years (n=65; 23 acute, 10 convalescent, 22 MIS-C and 10 naïve vaccinated), 5-11 years (n=71; 10 acute, 11 convalescent, 18 MIS-C and 32 naïve vaccinated) and 12-21 years old (n=77; 21 acute, 12 convalescent, 24 MIS-C and 20 naïve vaccinated). Mean antibody binding values are shown and are color coded by group. All SPR experiments were performed in duplicate and the researchers performing the assay were blinded to sample identity. The variations for duplicate runs of SPR were <5%. The data shown are average values of two experimental runs. The statistical significances between the variants were performed using R. The differences were considered statistically significant with a 95% confidence interval when the p value was less than 0.05. The significant p-values are shown.

Figure S4

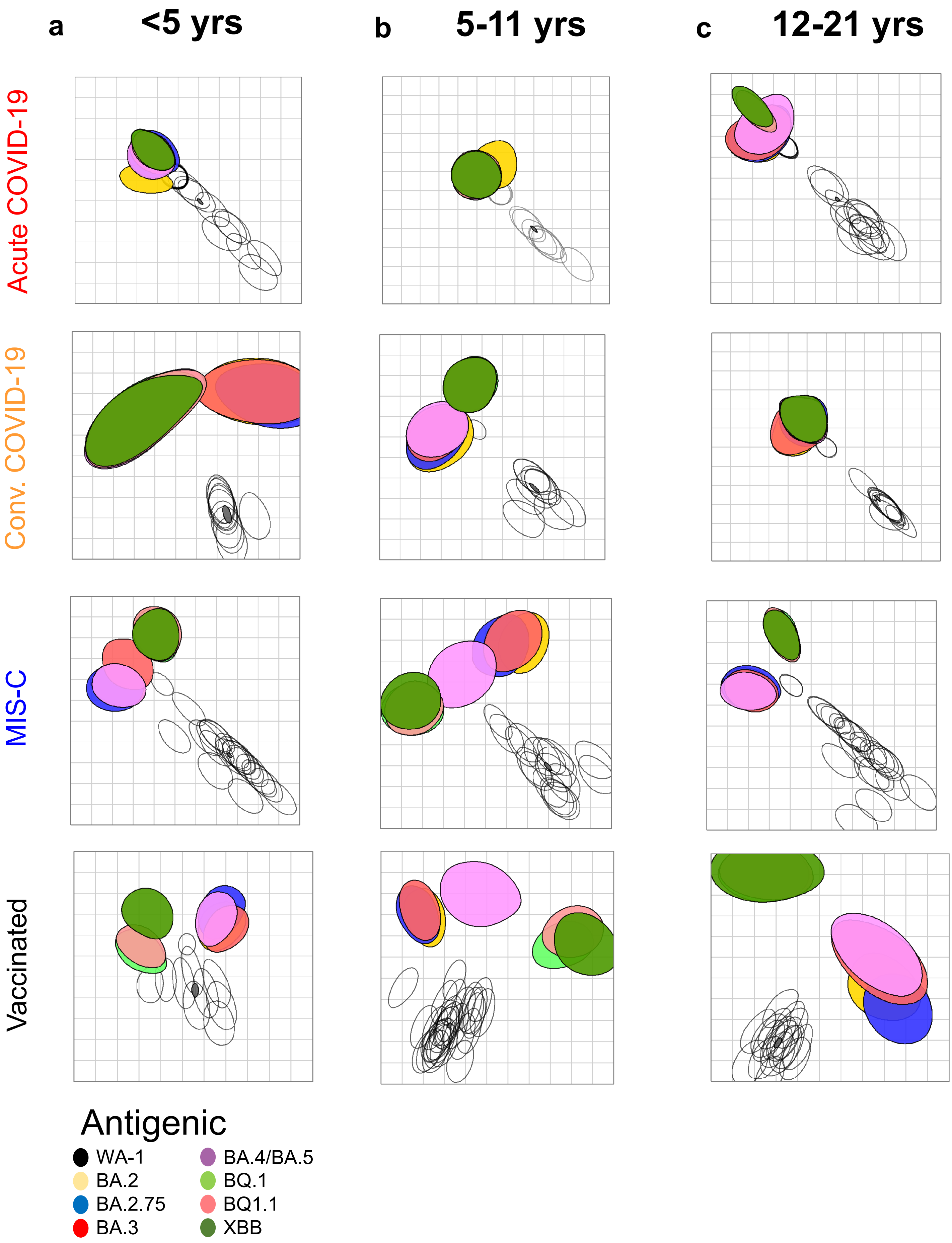
a
<5 yrs
b
5-11 yrs
c
12-21 yrs
Acute COVID-19
Conv. COVID-19
MIS-C
Vaccinated
Antigenic
WA-1
BA.2
BA.2.75
BA.3
BA.4/BA.5
BQ.1
BQ1.1
XBB

Figure S4: Antigenic cartography of acute COVID-19 vs convalescent COVID-19 vs MIS-C vs vaccination in different pediatric age groups. Antigenic maps were generated for younger children (<5 years in ‘a’), school-age children (5-11 years in ‘b’), and adolescent (12-21 years in ‘c’), with either acute COVID-19, convalescent COVID-19, MIS-C or vaccination, against SARS-CoV-2 WA-1 the Omicron subvariants. To assess the impact of uncertainty in titers and variant reactivity on the antigenic cartography map, a "smooth" bootstrap approach was performed using 1000 bootstrap repeats, with 100 optimizations per repeat. The standard deviation of the noise added to the neutralization titers was 0.7, while the standard deviation for the noise added to antigen reactivity was 0.7. The resulting antigenic map displayed colored regions representing the area capturing 68% (one standard deviation) of the positional variation for each serum or variant. Despite the noise added, both random and systematic, the antigenic relationships demonstrated robustness of the antigen and serum positions.

Figure S5

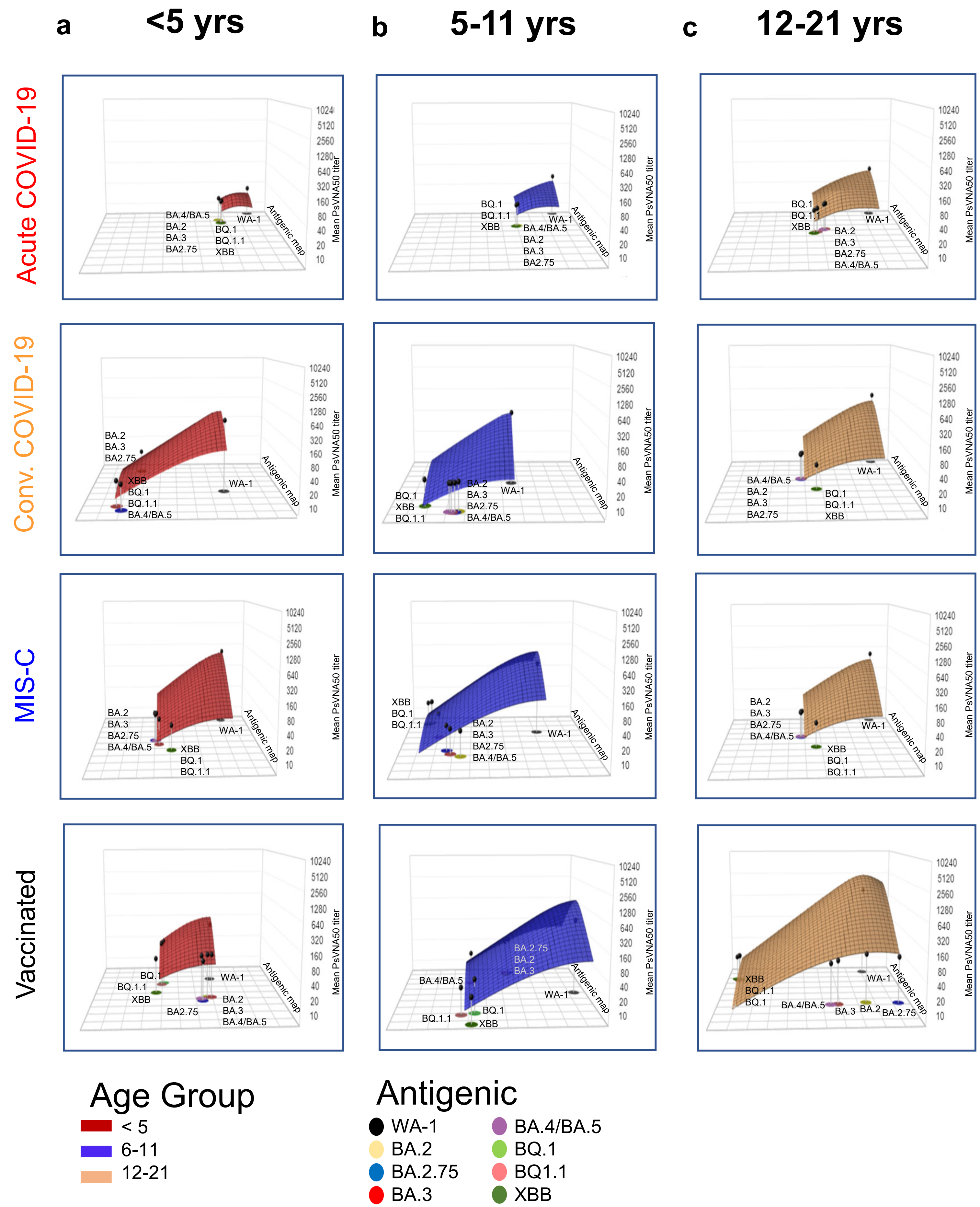

a
<5 yrs
b
5-11 yrs
c
12-21 yrs
Acute COVID-19
Conv. COVID-19
MIS-C
Vaccinated
Mean PsVNA50 titer
Antigenic map
Age Group
< 5
6-11
12-21
Antigenic
WA-1
BA.2
BA.2.75
BA.3
BA.4/BA.5
BQ.1
BQ1.1
XBB

Figure S5: Neutralizing antibody landscapes following infection or vaccination for different age groups of children. The antigenic landscape was generated using the SARS-CoV-2 neutralization titers against WA-1 and Omicron BA.2, BA.3, BA.2.75, BA.4/BA.5, BQ.1, BQ1.1 and XBB.1 for the 213 children with either acute COVID-19, convalescent COVID-19, MIS-C or following vaccination, divided by age categories: (a) <5 years (n=65; 23 acute, 10 convalescent, 22 MIS-C and 10 naïve vaccinated), (b) 5-11 years (n=71; 10 acute, 11 convalescent, 18 MIS-C and 32 naïve vaccinated) and (c) 12-21 years old (n=77; 21 acute, 12 convalescent, 24 MIS-C and 20 naïve vaccinated). The x and y axis of each landscape map represents antigenic cartography between WA-1 and variants, with colored points representing the locations of each SARS-CoV-2 strain. The grid squares (1 antigenic unit) correspond to a 2-fold change in neutralization assay. The average landscape for each serum group was constructed by fitting landscapes for each children serum sample, with the peak of the landscape indicating the mean PsVNA50 titers for that group against the respective SARS-CoV-2 strain on the z-axis. The landscapes are color coded according to age groups. The magnitude of neutralization titers (Mean PsVNA50 titer) for children either following vaccination or infection against each SARS-CoV-2 strain are shown by lines connected for each variant on the landscape.
