# Supplementary material for: Neutralization of SARS-CoV-2 Omicron BQ.1, BQ.1.1 and XBB.1 variants following SARS-CoV-2 infection or vaccination in children
Source: Nat Commun. 2023 Dec 1;14:7952. doi: 10.1038/s41467-023-43152-y (PMC10692185; doi:10.1038/s41467-023-43152-y)
Supplement: Supplementary file 3 — Reporting Summary [file 41467_2023_43152_MOESM3_ESM.pdf]

nature portfolio

Corresponding author(s): Surender Khurana

Last updated by author(s): Sep 22, 2023

Reporting Summary

Nature Portfolio wishes to improve the reproducibility of the work that we publish. This form provides structure for consistency and transparency in reporting. For further information on Nature Portfolio policies, see our Editorial Policies and the Editorial Policy Checklist.

Statistics

For all statistical analyses, confirm that the following items are present in the figure legend, table legend, main text, or Methods section.

n/a	Confirmed	
☐	☒	The exact sample size (*n*) for each experimental group/condition, given as a discrete number and unit of measurement
☐	☒	A statement on whether measurements were taken from distinct samples or whether the same sample was measured repeatedly
☐	☒	The statistical test(s) used AND whether they are one- or two-sided<br>*Only common tests should be described solely by name; describe more complex techniques in the Methods section.*
☐	☒	A description of all covariates tested
☐	☒	A description of any assumptions or corrections, such as tests of normality and adjustment for multiple comparisons
☐	☒	A full description of the statistical parameters including central tendency (e.g. means) or other basic estimates (e.g. regression coefficient) AND variation (e.g. standard deviation) or associated estimates of uncertainty (e.g. confidence intervals)
☐	☒	For null hypothesis testing, the test statistic (e.g. *F*, *t*, *r*) with confidence intervals, effect sizes, degrees of freedom and *P* value noted<br>*Give P values as exact values whenever suitable.*
☒	☐	For Bayesian analysis, information on the choice of priors and Markov chain Monte Carlo settings
☐	☒	For hierarchical and complex designs, identification of the appropriate level for tests and full reporting of outcomes
☐	☒	Estimates of effect sizes (e.g. Cohen's *d*, Pearson's *r*), indicating how they were calculated

Our web collection on statistics for biologists contains articles on many of the points above.

Software and code

Policy information about availability of computer code

Data collection	Antibody data was collected in MS Excel version 16.57.
Data analysis	Antibody titers were calculated using Prism 9.3.1 (GraphPad Software). Statistical analysis were performed using R statistical software (version 4.1.2), and all information is provided the statistical methods section.

For manuscripts utilizing custom algorithms or software that are central to the research but not yet described in published literature, software must be made available to editors and reviewers. We strongly encourage code deposition in a community repository (e.g. GitHub). See the Nature Portfolio guidelines for submitting code & software for further information.

Data

Policy information about availability of data

All manuscripts must include a data availability statement. This statement should provide the following information, where applicable:

- Accession codes, unique identifiers, or web links for publicly available datasets
- A description of any restrictions on data availability
- For clinical datasets or third party data, please ensure that the statement adheres to our policy

All data are shown in manuscript figures and supplementary information. The complete dataset for this study is provided in the Source Data file.

Research involving human participants, their data, or biological material

Policy information about studies with human participants or human data. See also policy information about sex, gender (identity/presentation), and sexual orientation and race, ethnicity and racism.

Reporting on sex and gender	All children irrespective of sex were eligible for the study. Sex was not considered in the study design and study findings apply to children of both sexes. The sex was determined based on self-reporting. The sex distribution in each cohort is shown in Supplementary Table S2. Of the total of 223 children, 112 self-reported as males. No sex-based analyses were performed in this study.
Reporting on race, ethnicity, or other socially relevant groupings	All children irrespective of their race or ethnicity were eligible for the study. Race or ethnicity was not considered in the study design and study findings apply to children of all race or ethnicities. The race and ethnicity was determined based on self-reporting. The race or ethnicity status in each cohort is shown in Supplementary Table S2. No race-based analyses were performed in this study.
Population characteristics	Participants in this study were aged 0-21 years old. All children irrespective of sex, race, ethnicity, or previous health status that were exposed to SARS-CoV-2 or COVID-19 vaccination were eligible for the study. The sex, race, ethnicity, and previous health status was self-reported. Sex, race, ethnicity, or previous health status was not considered in the study design and study findings apply to children of both sexes or any race or ethnicity. The sex distribution, race, ethnicity, and previous health status in each cohort is shown in Supplementary Table S2. No sex-based or race-based analyses were performed in this study.
Recruitment	All patients SARS-CoV-2 infection or naive SARS-CoV-2 vaccinees were eligible without any specific selection criteria. Samples were collected from children following informed consent and assent from at least one parent or legal guardian as appropriate for age to participate in the study during the pandemic. Pediatric participants were recruited in a Overcoming COVID-19 multicenter network study or the MGH or the BCH study without any selection bias.
Ethics oversight	Children and adolescents, 0 to 21 years of age, with confirmed SARS-CoV-2 positive PCR or antibody testing were enrolled into an IRB-approved specimen collection protocol following informed consent and assent as appropriate for age. The Overcoming COVID-19 Network studies severe complications of COVID-19 in children and adolescents consists of a subgroup of 20 sites in 18 U.S. states. Study sites relied on a single IRB at Boston Children's Hospital under Protocol Number #IRB-P00033157, and informed consent was obtained from at least one parent or legal guardian. Samples from the Boston Children's Hospital COVID-19 Biobank included consented patient samples and deidentified samples obtained with IRB approved waiver of consent.<br>Vaccinated healthy control samples from Mass General for Children and Boston Children's Hospital were obtained following parental and subject consent and assent as appropriate. The protocol was approved by the IRB at MassGeneral Brigham Institutional Review Board (IRB) (MGB #2020P000955).<br>Samples were tested in different antibody assays with approval from the U.S. Food and Drug Administration's Research Involving Human Subjects Committee (FDA-RIHSC) under exemption protocol '252-Determination-CBER-2020-04-02'.

Note that full information on the approval of the study protocol must also be provided in the manuscript.

Field-specific reporting

Please select the one below that is the best fit for your research. If you are not sure, read the appropriate sections before making your selection.

☒ Life sciences ☐ Behavioural & social sciences ☐ Ecological, evolutionary & environmental sciences

For a reference copy of the document with all sections, see nature.com/documents/nr-reporting-summary-flat.pdf

Life sciences study design

All studies must disclose on these points even when the disclosure is negative.

Sample size	All available samples were analyzed in this study
Data exclusions	No data was excluded
Replication	Neutralization and SPR were performed twice by independent researchers in the lab. The replications were successful. The variation in duplicate experimental runs was <10% for neutralization and <5% for SPR.
Randomization	All samples from the children were analyzed in this study. The study was non-randomized performed during the pandemic on outpatient and inpatients. Initially, no patient information was provided, and all the immune analyses were conducted blindly by the researcher's performing the assays. The participants were assigned in each experimental group based on their hospitalization status and clinical outcome.
Blinding	Experiments were performed by different investigators, who were blinded to sample identity.

Reporting for specific materials, systems and methods

We require information from authors about some types of materials, experimental systems and methods used in many studies. Here, indicate whether each material, system or method listed is relevant to your study. If you are not sure if a list item applies to your research, read the appropriate section before selecting a response.

Materials & experimental systems

n/a	Involved in the study
☒	☐ Antibodies
☐	☒ Eukaryotic cell lines
☒	☐ Palaeontology and archaeology
☒	☐ Animals and other organisms
☒	☐ Clinical data
☒	☐ Dual use research of concern
☒	☐ Plants

Methods

n/a	Involved in the study
☒	☐ ChIP-seq
☒	☐ Flow cytometry
☒	☐ MRI-based neuroimaging

Eukaryotic cell lines

Policy information about cell lines and Sex and Gender in Research

Cell line source(s)	293_ACE2_TMPRSS2 cell line was generated and sourced from the lab of Carol Weiss at FDA (Neerukonda, S.N. et al. (2021) PLoS One 16, e0248348).
Authentication	Cell line was checked for expression of ACE2 and validated by FACS analysis. The cell line was not authenticated by karyotyping or other genomic techniques. Reference: Neerukonda, S.N. et al. Establishment of a well-characterized SARS-CoV-2 lentiviral pseudovirus neutralization assay using 293T cells with stable expression of ACE2 and TMPRSS2. PLoS One 16, e0248348 (2021).
Mycoplasma contamination	Negative for Mycoplasma
Commonly misidentified lines (See ICLAC register)	No misidentified cell lines were used in the study.
